# Commuting in crosswinds and foraging in fast winds: the foraging ecology of a flying fish specialist

**DOI:** 10.1098/rspb.2025.0774

**Published:** 2025-08-06

**Authors:** Ruth E. Dunn, Alice M. Trevail, Malcolm A. C. Nicoll, Robin Freeman, Charles A. Braman, Bethany L. Clark, Charlotte Mitchell, Abigail W. Schiffmiller, Hannah Wood, Stephen C. Votier

**Affiliations:** ^1^ Lyell Centre, Heriot-Watt University, Edinburgh, UK; ^2^ Lancaster Environment Centre, Lancaster University, Lancaster, UK; ^3^ Centre for Ecology and Conservation, University of Exeter, Penryn, UK; ^4^ Zoological Society of London Institute of Zoology, London, UK; ^5^ Ecology, Evolution, and Marine Biology, UC Santa Barbara, Santa Barbara, CA, USA; ^6^ BirdLife International, Cambridge, UK; ^7^ Department of Biology and Wildlife, University of Alaska Fairbanks, Fairbanks, AK, USA

**Keywords:** red-footed booby, energetics, foraging costs, flight behaviour, GPS tracking, movement ecology, seabirds, tropical seabird

## Abstract

Understanding how the behaviour of volant species is influenced by winds is important at a time when global airflow patterns and intensities are shifting. We investigated how wind speeds and directions influenced the flight and feeding events of a flap-gliding seabird during central place trips searching for aerial prey like Exocoetidae flying fish. We deployed GPS accelerometers on red-footed boobies (*Sula sula rubripes*) in the Chagos Archipelago (Indian Ocean) for 45 foraging trips. By comparing foraging commutes to simulated alternative routes, we demonstrate that birds preferentially selected tailwinds and crosswinds, with stronger selection during the outbound compared with the inbound leg. By selecting favourable winds, birds reached higher ground speeds without having to increase flapping flight. Selecting favourable wind conditions may be an adaptation to tropical pelagic habitats and ephemeral prey. Hidden Markov models, used to characterize behavioural states, revealed that birds were more likely to forage during windier conditions, perhaps aided by increased accessibility of flying fish—which a small sub-sample of bird-borne video cameras revealed were largely caught on the wing. We therefore show how wind has divergent consequences for foraging journeys and feeding events, with implications for understanding the ecological effects of climate change-driven wind alterations.

## 1. Introduction

The Earth’s climate is changing at an unprecedented rate, influencing its weather systems, ocean currents and wind regimes [1]. Shifts in the patterns and intensities of winds are particularly likely to impact volant species, with potential repercussions for their behaviour, distributions and demography [2–5]. Understanding the influence of wind on flight and foraging behaviour is therefore vital if we are to predict the sensitivity of different species to changing environmental conditions [6,7].

The effects of wind on a flying animal vary with its ecology, flight style and local environments. For example, many migratory species, from soaring raptors to high-flying moths, select specific winds to aid them in undertaking energy-efficient migrations across land and sea [8,9]. In addition to harnessing beneficial winds when undertaking some of the world’s longest migrations [10,11], soaring albatrosses and shearwaters also exploit crosswinds and wind speed gradients close to the ocean’s surface while performing long central-place foraging trips [12,13]. Though the flights of some species (e.g. Procellariiformes) benefit from high wind speeds, enabling them to dynamic soar [2], for those that propel themselves via intensive flapping flight (e.g. Alcidae), strong winds (particularly headwinds) can induce high energetic costs [14,15]. Currently, a mechanistic understanding of whether flap-gliding seabirds (central-place foragers that alternate between short periods of flapping and gliding flight) select for specific wind directions and speeds, and how any selection might influence their travel, remains limited.

In addition to impacting flight, wind can also affect species’ foraging efficiencies via its influence on predator behaviour as well as the detectability and accessibility of prey [16]. For example, the predation success of European sparrowhawks (*Accipiter nisus*) is lower in high wind speeds due to air turbulence disrupting their flight control [17]. Within the marine environment, both wind and wave conditions may have a wide range of complex effects on flying seabird foraging behaviour. For instance, wind and waves can interact and influence the behaviour of visually foraging, aerial-diving seabirds, restricting them to target prey close to the water’s surface [18] or reducing prey capture rates via impacts on their ability to locate or track prey from the air [19]. Furthermore, inclement weather and increased wave action, associated with high winds, can also hinder prey capture via increased turbidity [20], or by driving prey to deeper depths [21]. Contrastingly, when blowing in certain directions, high winds and increased wave action can enhance the upwelling of nutrient-rich waters, leading to subsequent increases in primary productivity, the attraction of higher trophic level species and increased seabird feeding opportunities [22]. They may also aid navigation of the ocean surface’s odour landscape, crosswind flight enabling some procellarid seabirds to maximize the use of their sense of smell, locate odour plumes and capture prey [23,24]. Finally, wind and wave conditions may also impact the behaviour of aerial prey such as Exocoetidae flying fish and Ommastrephidae squid, which are important food for many tropical seabirds. These prey species are efficient gliders [25] which may engage in longer flights when supported by updrafts, created by strong winds [26] blowing over large waves [27], and thus be more accessible to species which catch them on the wing [28]. Nevertheless, how wind and wave conditions influence the decisions of seabirds to engage in foraging behaviour within tropical systems remains to be explored.

Red-footed boobies (*Sula sula rubripes*; hereafter ‘RFB’) are a widespread tropical seabird that breed across the Atlantic, Indian and Pacific Oceans between 23° N and 23° S, where they are thought to take advantage of wind energy to reduce their own energetic costs during flight [29]. While breeding, RFBs use flap-gliding flight to commute hundreds of kilometres to their pelagic foraging grounds [30–32] where they feed by aerial capture or shallow plunge diving for prey like flying fish [33], before then performing fast, direct journeys back to their breeding colonies [34]. The availability of productive feeding areas within the tropical oceanic ecosystems that RFBs inhabit may only be spatially predictable for short periods [35,36], being driven by transient processes, such as ephemeral fronts and irregular eddies, or via facultative multi-species associations [37]. Here, we used GPS, acceleration and video data from loggers deployed on RFBs to explore how, in lieu of predictable foraging locations, wind impacts both their foraging journeys as well as their feeding events. Firstly, we sought to investigate wind selectivity during the outbound and inbound stages of the foraging commute. While soaring seabirds such as shearwaters select cross-tailwinds to perform energetically efficient journeys [5], species with predominantly flapping flight such as black-legged kittiwakes (*Rissa tridactyla*) do not appear to preferentially select certain winds [38]. We therefore predict that for flap-gliding species such as RFBs, there are likely benefits of selecting tailwinds and crosswinds at intermediate wind speeds (though crosswinds may be less favourable than they are for Procellariiformes), and that birds will avoid strong headwinds [32,39]. Secondly, we investigate how wind impacts *en route* ground speeds and flapping flight (as a metric for the cost of travel), providing us with a better understanding of the mechanistic reasons for selecting certain wind conditions. We predict that ground speeds will be highest and the proportion of flapping flight lowest in preferable tailwind and crosswind conditions [40]. Finally, we investigated how wind and wave conditions influenced feeding probability by identifying behavioural transitions between feeding, travelling and resting, using bird-borne video to also determine prey capture behaviour (i.e. whether by diving or capture in flight). Wind probably aids flying fish flight, allowing them to glide, and therefore be exposed above the water’s surface for longer [26]. This prey type may therefore be more accessible at the water’s surface in high winds, and we predict that birds might preferentially feed in such conditions.

## 2. Methods

### (a) Data collection

Fieldwork was conducted on islands within the Chagos Archipelago Marine Protected Area, central Indian Ocean, which hosts over 21 000 breeding pairs of RFB that largely remain within the protected area throughout the year [41]. We caught shrub-nesting adults on the nest by hand or with a landing net, selecting individuals that were incubating eggs (January 2024) or brooding chicks (February 2019 and 2022). We captured 15 individuals at Barton Point (7.23° S, 72.43° E; 9269 breeding pairs) in February 2019, 17 individuals at East Island (7.23° S, 72.42° E; 1113 breeding pairs) in February 2022 and 6 individuals at Nelsons Island (5.69° S, 72.31° E; 3300 breeding pairs) in January 2024.

We attached 18 g TechnoSmArt Axy-Trek Marine data loggers (comprising 1.7–2.4% of body mass) on the underside of each bird’s central two to four tail feathers, depending on moult condition. The loggers had GPS antennas, tri-axial accelerometers, pressure sensors and temperature loggers. In 2024, we also attached video cameras to the birds’ central back feathers (DVL400M, Little Leonardo co., an additional 15 g, representing a further mean 1.7% of body mass across the five individuals where mass was taken on their deployment). In 2019 and 2022, geolocation and immersion loggers (Intigeo C330, Migrate technology, 3.3 g, representing a mean of 0.4% of body mass) were also attached to the birds via a plastic leg ring, but the resultant data from these loggers were not analysed in this study. We used marine Tesa tape (4651) for all attachments. Handling times lasted approximately 10 min in 2019 and 2022, and 15 min in 2024, which included bird weighing and measurements. Nest attendance was monitored every 1−3 days following deployment, revealing no unusual behaviour, and that disturbance caused during the deployment process was short-lived. Individuals were recaptured after at least 36 h of logger deployment, when they were assumed to have completed at least one foraging trip. Devices were removed upon retrieval with recovery rates varying among sites, due to variation in access (73% at Barton Point, 52% at East Island and 83% at Nelson’s Island). We recovered 33% of video cameras, with losses attributable to long foraging trips during incubation. Birds regurgitated on recovery indicating that they had fed successfully.

All seabird tracking was approved by ethics committees at the Institute of Zoology, Zoological Society London, the University of Exeter and Heriot-Watt University under licence from the British Trust for Ornithology.

### (b) Data processing

We performed all data processing and analyses in R version 4.4.1 [42], unless stated otherwise.

#### (i) Location data

Technical faults in the GPS loggers in 2024 meant irregular location estimates, and we therefore focused on the 2019 and 2022 data for our locational analyses. In 2019, data loggers recorded GPS locations (latitude and longitude) every 30 s, and in 2022, they recorded bursts of 1 Hz GPS data for 15 s every 5 min. We used the ‘ExMove’ toolkit to subset our data to only include data from RFB foraging trips (i.e. periods when GPS locations extended more than 1 km from the colony for more than 30 min [30,43]). To regularize the GPS data and account for occasional gaps, we estimated locations at regular 5 min time intervals by fitting a state-space model using a correlated random walk model with the ‘aniMotum’ R package [44]. We categorized the outbound, middle and inbound stages of each foraging trip by using distance thresholds [12]. We therefore defined the middle of each foraging trip as the locations where the distance from the colony was at least 75% of the maximum distance travelled (electronic supplementary material, figure S1). The outbound and inbound stages of the trip were defined as the periods prior to and following the middle stage of the trip, respectively (electronic supplementary material, figure S1).

#### (ii) Video data

**Figure 1.**
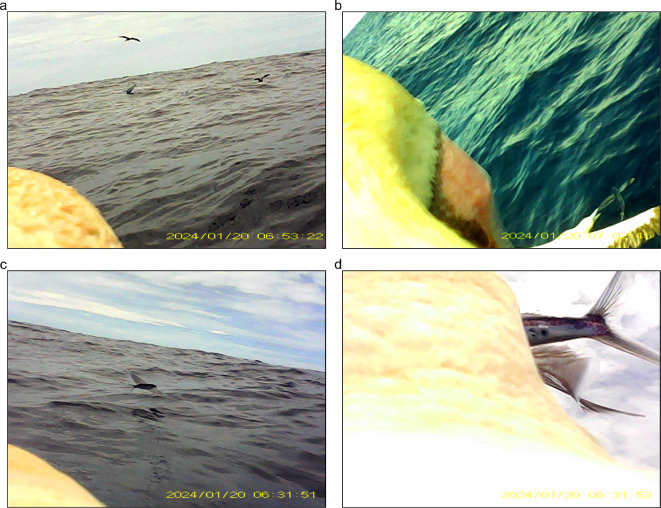
Bird-borne video camera footage of a red-footed booby *Sula sula rubripes* (a) flying close to a yellowfin tuna (*Thunnus albacares*) as it breaches the water’s surface, (b) foraging near a conspecific that is catching an exocoetid flying fish, (c) chasing a flying fish and (d) capturing the flying fish. The time is shown in UTC, whereas the local time in the Chagos Archipelago is UTC+5.

Bird-borne video cameras deployed in 2024 were programmed to turn on at approximately 11.00 local time the day after deployment and to then film continuously until the battery was exhausted. We used BORIS video coding software [45] to classify the following behaviours from the video footage: flight (including both flapping and gliding flight), beginning to dive towards the water’s surface, underwater, sitting on the water’s surface and taking off from the water into flight. These classifications were used to label behaviours within the acceleration data (see below). We also made observations of multi-species (figure 1a) or multi-individual foraging interactions (figure 1b) and prey capture events (figure 1c–d) in the video footage, but due to the sparsity of occurrence, these events were not the focus of this study.

#### (iii) Acceleration data

The tri-axial accelerometers recorded acceleration at 25 Hz in the three axes: surge (X), sway (Y) and heave (Z). We corrected for differences in device orientation by rotating these acceleration data using the ‘tagtools’ R package [46], standardizing the surge and sway axes to around 0 g and the heave axis to around 1 g. We were only able to calibrate the timestamps between the footage collected from one bird and its associated GPS and acceleration logger, and for this bird, we then used Framework4 software [47] to manually label the acceleration data that coincided with the video footage into the previously described behaviours. We used the knowledge gained from labelling the acceleration data that aligned with the approximately 3 h of video data from 1 bird in 2024 to inform labelling of approximately 50 incidences, distributed along the entire foraging trip, of the following behaviours for a further 10 individuals: flapping flight, gliding flight, diving (including both diving towards the water’s surface as well as time spent underwater due to the short durations of these events), sitting on the water’s surface and taking off from the water’s surface.

We extracted the first 1 second (25 records) of each labelled section of acceleration data to form a training dataset [48]. For each of these sections we calculated the mean ODBA (overall dynamic body acceleration), mean VeDBA (vectorial dynamic body acceleration), the mean and standard deviation of the roll (side-to-side movement) and pitch (vertical body angle), the mean, standard deviation, minimum and maximum of the surge, sway and heave axes, and the mean temperature (recorded every 1 s, potentially indicative of the logger being wet/dry) [48]. We used this training data within a supervised machine learning algorithm, using the ‘randomForest’ R package [49]. The Random Forest algorithm performs well at determining behaviour from acceleration data [50] and is capable of using training data from a small number of individuals and then transferring this to apply to a larger sample [51]. We used 10 000 trees per model, randomly sampled five metrics for each node within a tree, and used the resultant model to predict the behaviour of every 1 s segment of each individual’s foraging trips. Random forest models take a random sample of variables at each node within an individual decision tree to prevent overfitting and so the use of 10 000 trees in combination to make the final prediction meant that all variables would be used in many of the trees and taken into account equally. The model identified 98.8% of flapping flight, 92.1% of gliding flight, 85.7% of diving, 95.1% of sitting and 99.7% of take-off segments correctly, when compared to the labelled training dataset. We then applied this model over the full dataset. Following visual inspections of the predictions, we then also used logic-based corrections to reassign labels that were contextually incorrect, whereby for every <1 s instance of sit, take off or dive that occurred among segments that were otherwise labelled as flight, we adjusted these labels to also be flight [52].

#### (iv) Environmental variables

We obtained hourly 0.25° spatial resolution wind and wave data from the ‘ERA5 global atmospheric reanalysis’ dataset, the fifth generation European Centre for Medium Range Weather Forecasts (ECMWF) reanalysis models of global climate and weather [53]. From here, we downloaded individual wave height, wave periodicity and mean wave direction data, as well as zonal and meridional wind components, which were used to calculate wind speed and direction [54]. For each interpolated GPS location, we extracted wind and wave data that were closest in time using the ‘raster’ package [55]. We then also calculated wind and wave direction relative to bird movement, scaled to between 0° (tailwinds/receding waves) and 180° (headwinds/oncoming waves), for each location [56].

### (c) Statistical analyses

#### (i) Wind selectivity during outbound and inbound foraging commutes

**Figure 2.**
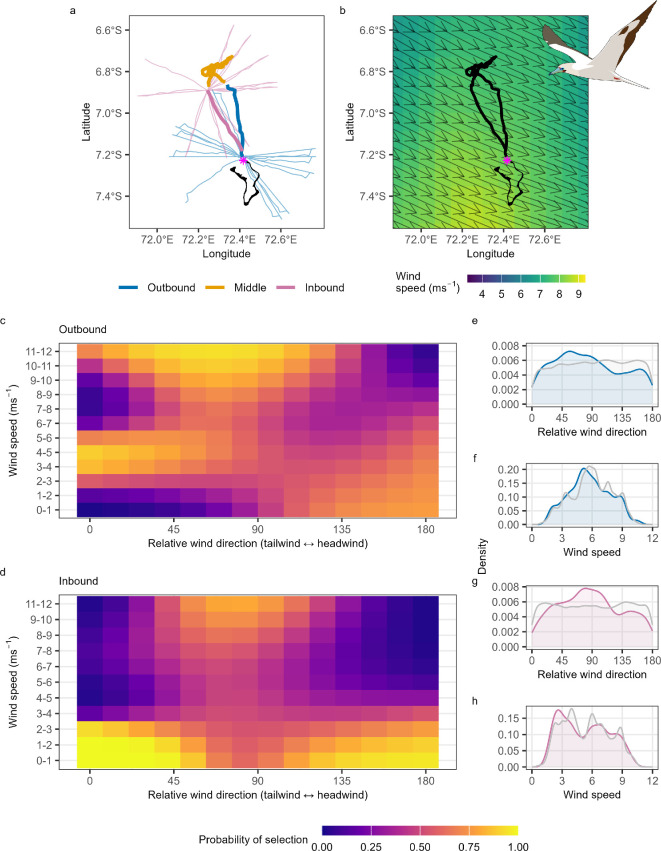
Determinants of wind selectivity by red-footed boobies *Sula sula rubripes* during their commuting flights. Map illustrating a single example foraging trip from East Island, Diego Garcia, Chagos Archipelago (a) coloured by trip stage (outbound, middle, inbound) with randomly rotated trips (*n* = 10, selected via AUC comparisons) shown as finer lines, and (b) overlaid on the average wind speeds and directions (arrows) experienced during the trip (bird ID = GY02207, year = 2022). The location of the colony is indicated via pink asterisks. (c) Heatmap of the probability of wind selectivity across all outbound stages of all foraging trips, given relative wind direction and wind speed (model AUC = 0.68). (d) Heatmap of the probability of wind selectivity across all inbound stages of all foraging trips, given relative wind direction and wind speed (model AUC = 0.71). Model predictions for panels c and d are provided within electronic supplementary material, S4. Density plots of the used (blue = outbound; pink = inbound) and available (grey) wind conditions, namely, (e) relative wind direction and (f) wind speed during the outbound commute and (g) relative wind direction and (h) wind speed during the inbound commute.

To investigate wind selectivity during the outbound and inbound stages of each foraging trip, we compared observed trips with up to 20 simulated journeys generated by rotating each outbound and inbound leg by 20 randomly selected angles varying between 0° and 360° [57] (figure 2a), using the ‘map’ function from the ‘purrr’ R package [58]. Following the methods outlined above, we extracted wind speed and relative wind direction from each simulated GPS location (figure 2b).

We modelled the probability of a route being selected (i.e. ‘route selectivity’) within a Bayesian generalized additive model, where the response variable was determined via a Bernoulli distribution (0 = simulated locations and 1 = original locations, weighted according to their relative proportions in the dataset). Visualizations of the environmental variables revealed that wind speed, wave height and wave periodicity were collinear and so we restricted these analyses to the explanatory variables of relative wind direction and wind speed (which were not collinear). We centred and standardized these variables before including them within a smooth interaction term, modelled using a tensor product spline, restricted to four knots, allowing us to model potential nonlinear interactions.

We included a unique identifier for each individual as a random effect, and a unique identifier for each foraging trip nested within the individual identifiers to account for potential non-independence among GPS fixes from the same trip and the same individual. The models were run with weakly informative priors for 4 chains, each with 2000 iterations, and a warmup of 1000 using the ‘brms’ R package implemented in STAN [59]. We determined the ratio of simulated locations per original location by running our models with an increasing number of available locations, determining what number was best to include by comparing the models’ area under the receiver operator characteristic curve (AUC) values (optimal number = 10) [60]. Model convergence and fit were assessed by inspecting posterior predictive plots, trace plots, effective sample sizes and the Gelman–Ruban convergence diagnostic, $\hat{R}$ [61]. We also assessed model validity via their AUC, with values of greater than 0.5 indicating that the model discriminated better than chance, and values of greater than 0.7 indicating useful applications [62].

#### (ii) Wind effects on commuting flight behaviour

For every commuting GPS fix that occurred during the outbound or inbound stage of the trip, we calculated the proportion of time spent in flapping flight (as opposed to gliding flight), as well as the mean ground speed. We investigated the influence of wind conditions on these metrics by again fitting Bayesian generalized additive models that were run and assessed using the same predictor variables, random effects structures and methods outlined above. Wave height was excluded from this analysis due to its collinearity with wind speed. We assumed that the proportion of time in flapping flight was determined by a Beta distribution and that ground speed was parametrized by a Gaussian distribution.

#### (iii) Impact of wind and waves on behavioural state probabilities

Using the interpolated GPS data across the entirety of all the foraging trips, we assessed whether stationary probabilities (‘state occupancy’) of remaining in at-sea behavioural states (feeding, travelling, resting) were influenced by wind speed, relative wind direction and relative wave direction. We also assessed the influence of these covariates on transition probabilities between behavioural states (electronic supplementary material, figure S2). To do this, we fitted hidden Markov models (HMMs) with the ‘momentuHMM’ R package [63], classifying the states via two input variables: step length (parametrized by a gamma distribution) and turning angle (parametrized by a von Mises distribution). We attempted to identify three at-sea behavioural states (feeding, travelling, resting), as has previously been done via a similar method for this population [34]. When specifying initial input values, we initially ran simplified covariate-free HMMs with the ‘moveHMM’ package [64], choosing values randomly from within a range of biologically realistic values 25 times and then determining the most appropriate ones as those closest to the most frequently estimated. We then used these initial input values within a series of ten ‘momentuHMM’ models, including all combinations of wind speed, relative wind direction and relative wave direction, including two-way interactions between wind speed and relative wind direction, and wind speed and relative wave direction [56]. Model selection to identify the best supported model was based on Akaike’s information criterion (AIC). The Viterbi algorithm was used to estimate the most likely sequence of movement states under the fitted model, and this model was assessed for goodness of fit and autocorrelation via QQ, pseudo-residual and autocorrelation plots [64]. Annotated GPS fixes and tracks were plotted and visually assessed to confirm that the behavioural states assigned by the model matched the expected spatial patterns of the behaviours (i.e. more distal GPS fixes over straight trajectories corresponding to flight; tortuous paths between more clustered GPS fixes corresponding to foraging, and more clustered GPS fixes over straight trajectories corresponding to resting).

## 3. Results

During 2019 and 2022, we recorded 45 foraging trips across 18 individuals. In 2024, we obtained 395 min of bird-borne video footage from two individuals with four birds regurgitating during logger deployment and retrieval (four regurgitates were primarily flying fish and two included flying squid). Within the 145 min of footage during which visibility was good enough to identify travelling and feeding behaviours, we observed 15 prey capture attempts. Of these attempts, 14 involved aerial pursuit of prey (e.g. figure 1c–d), and one was a sub-surface dive.

### (a) Wind selectivity during outbound and inbound foraging commutes

During their foraging trips, birds experienced wind speeds of 0.5–11.8 m s^−1^ primarily from the southwest in 2019 (210–260°), and primarily from the northwest in 2022 (310–340°). Bird departure angles to the distal point varied throughout 0–360° across both years (electronic supplementary material, figure S3).

During outbound commutes (AUC = 0.68), route selectivity was influenced by wind speed (mean effect of smooth = 0.59, lower and upper credible intervals (CIs) = 0.55, 0.63), relative wind direction (mean effect of smooth = 0.54, CIs = 0.51, 0.58) and their interaction (mean effect of the smoothed interaction term = 0.56, CIs = 0.52, 0.6; figure 2c). In high wind speeds (7–12 m s^−1^), they flew with crosswinds (around 90° relative wind direction; figure 2c). Birds had a preference for tailwinds (around 0° relative wind direction) during intermediate wind speeds (2–6 m s^−1^; figure 2c), selected headwinds in very low winds (0–4 m s^−1^; figure 2c) and avoided them at high winds (8–12 m s^−1^; figure 2c).

During inbound commutes (AUC = 0.71), route selectivity was influenced by wind speed (mean effect of smooth = 0.53, CIs = 0.48, 0.59), relative wind direction (mean effect of smooth = 0.38, CIs = 0.24, 0.32), and their interaction (mean effect of the smoothed interaction terms = 0.46, CIs = 0.41, 0.51; figure 2d). Birds generally selected crosswinds in higher wind conditions (8–12 m s^−1^; figure 2d), showed limited selectivity during intermediate wind speeds (3–8 m s^−1^; figure 2d), and selected either tailwinds or headwinds at low wind speeds (0–3 m s^−1^; figure 2d).

### (b) Wind effects on commuting flight behaviour

**Figure 3.**
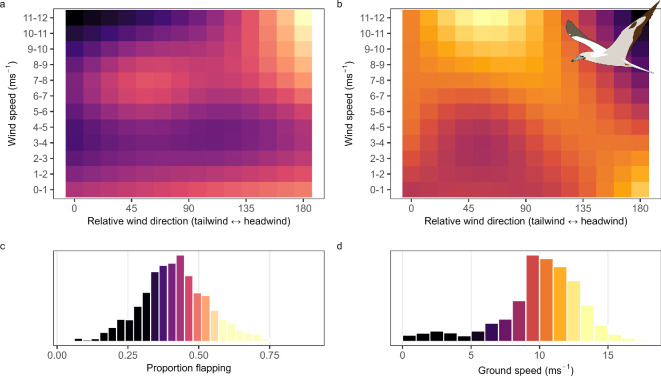
Influence of wind conditions on red-footed booby*Sula sula rubripes* flight. (a) Heatmap of the influence of the interaction between relative wind direction and wind speed on the proportion of time spent flapping, and (b) heatmap of the influence of the interaction between relative wind direction and wind speed on ground speed. Histograms of the (c) frequency of the proportion of time spent flapping, and (d) frequency of ground speeds. Model predictions are provided within electronic supplementary material, S6.

The outbound and inbound commutes comprised 41.7 ± 12% flapping flight, characterized by high maximum values and standard deviations in the heave axis, as well as high VeDBA and ODBA (electronic supplementary material, figure S5). The proportion of flapping flight was influenced by the nonlinear interaction between wind speed and relative wind direction (mean effect of the smoothed interaction term = 0.43, CIs = 0.4, 0.47) as birds flapped more in headwinds than crosswinds, and slightly more in crosswinds than tailwinds, especially in higher wind speeds (figure 3a).

Mean ground speed was 10 ± 3.1 m s^−1^, being influenced by a non-linear interaction between wind speed and relative wind direction (mean effect of the smoothed interaction term = 10.1, CIs = 9.2, 11.1; figure 3b). Ground speeds increased slightly with wind speed when flying in tailwind conditions, peaked with high wind speeds in crosswinds, and decreased with windspeed in headwind conditions (figure 3b).

### (c) Impact of wind and waves on behavioural state probabilities

The HMM categorized three at-sea behavioural states: ‘feeding’ characterized by short step lengths and wide turning angles (step length: 0.7 ± 0.8 km; turning angle: *μ* = −0.03, *κ* = 0.7), ‘travelling’ characterized by long step lengths and narrow turning angles (step length: 3.1 ± 0.6 km; turning angle: *μ* = −0.0008, *κ* = 19.2), and ‘resting’ characterized by short step lengths and narrow turning angles (step length: 0.1 ± 0.04 km; turning angle: *μ* = 0.003, *κ* = 25.2). Behavioural transitions varied according to a two-way interaction between relative wind direction and wind speed, as well as a two-way interaction between relative wave direction and wind speed (AIC = 30 698, ΔAIC of 29 compared to next best model).

**Figure 4.**
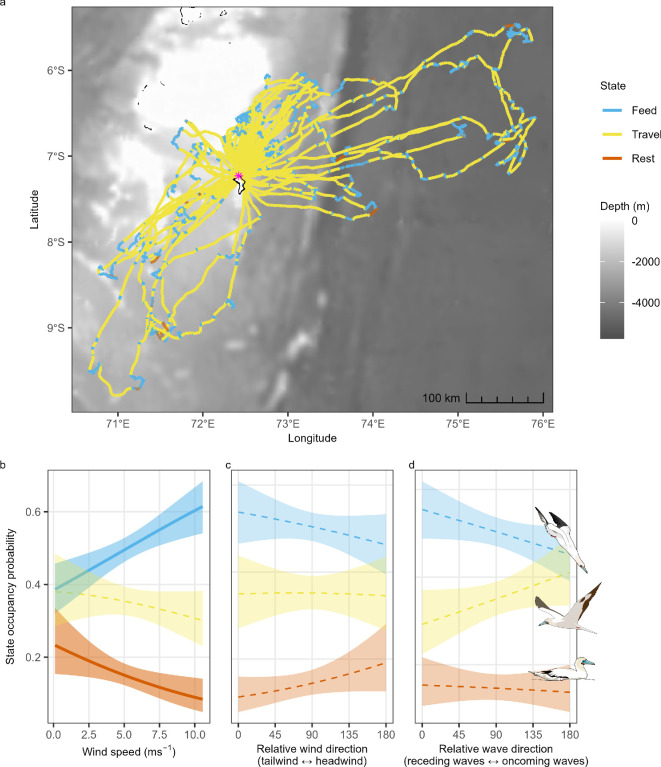
Influence of wind and wave conditions on the behavioural states (feeding, travelling, resting) of red-footed boobies *Sula sula rubripes* during their foraging trips. (a) Red-footed booby foraging trips from Diego Garcia (indicated via a pink asterisk), coloured by hidden Markov model (HMM) estimated behavioural state. HMM estimated state occupancy probabilities in relation to (b) wind speed, (c) relative wind direction and (d) relative wave direction. Lines indicate estimated means of the posterior predictive distributions, bracketed by 95% confidence intervals (shaded areas). Significant covariate effects, as determined by confidence interval overlap, are illustrated via thicker, solid lines, with darker shaded areas.

RFBs spent varying proportions of their foraging trips resting (0.15, CIs = 0.11, 0.19), travelling (0.34, CIs = 0.3, 0.41) and feeding (0.5, CIs = 0.46, 0.55; figure 4a). The probabilities of remaining in each of the behavioural states were high (feeding = 0.91, travelling = 0.92, resting = 0.88) and transition probabilities are described in electronic supplementary material, S2. Stationary state probabilities indicated that birds were more likely to continue to feed and less likely to remain in a state of rest in high wind speeds (figure 4b), with little influence of relative wind or wave directions on state occupancy (figure 4c–d). Travel state occupancy was also not influenced by wind speed, relative wind direction, or relative wave direction (figure 4b–d).

## 4. Discussion

Our multi-scale analyses demonstrate how winds shape both the commuting and feeding behaviour of a wide-ranging tropical seabird while central place foraging to feed primarily on flying fish. First, we demonstrate strong selection for cross/tail winds during the outbound commute, and crosswinds while returning to the colony. We then illustrate that wind selectivity was shaped, to an extent, by the costs and benefits of flapping flight and ground speed. Finally, we show that birds are more likely to feed in windy conditions—consistent with video footage of aerial flying fish capture. We thereby demonstrate that winds and waves can influence seabird foraging at multiple spatiotemporal scales, effecting both habitats and behaviours.

### (a) Wind selectivity during outbound and inbound foraging commutes

There were differences in wind selectivity between outbound and inbound journeys. During their outbound commutes, RFBs selected to fly with both crosswinds and tailwinds, although selection was limited at intermediate windspeeds, and headwinds were used in low wind speeds (figure 2c). During the inbound leg, birds generally selected crosswinds, with limited evidence for selectivity at low wind speeds (figure 2d). These differences are likely because the outbound flight has no fixed goal providing greater scope for selecting favourable winds [5]. Contrastingly, during inbound commutes, when birds are heading for the colony, while they could select less direct journeys to track favourable winds, longer and more time-consuming journeys may only feature under more extreme, stormy weather conditions [65].

During their outbound journeys, RFBs are driven by the obligation to find food. Birds may therefore be expected to favour commuting to learned, profitable foraging areas [66] instead of flying with the most advantageous wind conditions [67]. However, while RFB departure directions tend to be similar on successive trips [31], they exhibit low feeding site fidelity within the Indian Ocean [68] due to the often ephemeral nature of their prey, though this can vary by location and with oceanography [69]. Wind selectivity may therefore be a response, in part, to ephemeral feeding opportunities and the need to maintain low transit costs while searching for food. Moreover, RFBs in this population select foraging routes independently of oceanographic conditions (but not foraging location [33]).

Compared with outbound commutes, the return legs of RFB foraging trips are straighter and faster, presumably driven by the navigational certainty of heading homeward [34]. There may also be an additional imperative with birds travelling more quickly close to nightfall to mitigate potential at-sea predation risk [70]. Nevertheless, RFBs showed flexibility in their return trips, harnessing crosswinds (figure 2d) to fly at high ground speeds (figure 3c). How inbound commute wind selectivity might be complemented by other factors, such as the navigational and energetic advantages of commuting in echelon flight formations among conspecifics [71], remains to be determined.

### (b) Wind effects on commuting flight behaviour

We found that by selecting high-speed crosswinds and tailwinds, RFBs could fly swiftly while also gliding more than flapping (figure 3). RFBs have long, narrow wings relative to their body size, and resultant low wing-loading allowing them to glide quickly and efficiently in high crosswinds, minimizing their energetic expenditure during flight [29,67]. Within our study, however, RFBs avoided flying into strong headwinds as such conditions required energetically costly, flapping-intensive flight that did not realize the ground speeds reached when flying with crosswinds and tailwinds (figure 3).

### (c) Impact of wind and waves on behavioural state probabilities

Strong winds can negatively impact seabird foraging. For example, stomach temperature loggers, used to quantify ingestion, revealed that in stormy conditions (i.e. strong winds and rain) two albatross species have reduced prey capture rates, possibly because their prey (krill, non-flying fish and squid) are deeper or less visible within the water column [72]. Contrastingly, in high winds RFBs were more likely to continue feeding, and less likely to rest (figure 4b). Though we were not able to infer feeding success from our data, we speculate that RFBs may benefit from feeding in windy conditions, in part because their preferred prey (flying fish and squid) can glide for longer (up to a maximum of 400 m) in strong winds [26,73]. Indeed, for human fishers, catch rates of *Hirundichthys affinis* flying fish are positively associated with wind speed and swell height [27], wind- and wave-driven currents and upwellings improving flying fish foraging habitats [74]. Furthermore, the low aspect ratio of RFBs (even relative to other sulids [75,76]) may be an adaptation that aids manoeuvrability and turning performance in order to pursue and capture flying fish on the wing. More copious video data, collected in conjunction with GPS and accelerometry data, would be likely to reveal the mechanisms that cause differing wind conditions to benefit the foraging behaviour of flying fish specialists (e.g. do RFBs sustain their chase behaviour in high winds due to longer flying fish glides?).

When feeding, RFBs perform frequent dives, landings and take-offs [32,33], the latter incurring high energetic costs [77,78]. Wandering albatrosses (*Diomedea exulans*) are more likely to harness the energetic benefits of taking-off into strong headwinds [56], with the effort required for them to take off also being lower in wavier conditions [79]. Within this study, wind speed, wave height and wave periodicity were collinear and so we were not able to investigate their discrete effects; however, our observed interaction between wind speed and relative wave direction (electronic supplementary material, figure S2) indicates that both ocean waves and winds likely influence the decisions made by foraging RFBs.

### (d) Wider implications of wind effects

As global air flow patterns change, what are the likely consequences for flying seabirds? Higher winds at-sea could be advantageous for wide-ranging soaring species during their commutes [2], but the energetic costs or benefits will also depend on whether birds can optimize the use of tail and cross winds when feeding. For tropical seabirds, an increasingly windy marine realm may increase flying fish capture success, as is the case for gillnet flying fish fisheries [27].

While there is considerable uncertainty regarding changing wind regimes*—*many models suggest global ‘stilling’ [80]—one of the more consistent predicted effects is the increased frequency and intensity of storms [1]. The RFBs tracked in this study experienced winds up to a maximum of approximately 12 m s^−1^, far calmer conditions than gale force winds (>16 m s^−1^), for example. Other research has shown that RFBs seem resilient to storms, being able to forage effectively in gale force winds in contrast to frigatebirds at the same site in the Western Indian Ocean that are lighter and less well waterproofed [81]. However, while we show that RFBs can harness higher winds to their advantage, their behavioural responses are unlikely to be linear under projected increases in cyclone events (wind speeds >18 m s^−1^) and poleward shifts in trade winds [82]. For Indian Ocean albatrosses, changes in winds have already altered migration patterns [7], and also threaten increased foraging costs [2]. Understanding the perhaps divergent responses of seabirds to similar changes at lower latitudes, where different modes of prey capture are used, is both timely and vital.

## 5. Conclusion

By combining bird-borne GPS and acceleration data with environmental (wind and wave) data, we reveal how winds shape the behavioural decisions of RFBs at multiple scales, impacting both their foraging commutes and their feeding behaviour. Our bird-borne video reveals the prevalence of flying fish aerial capture and thus how wind may impact this specialist mode of foraging that is widespread throughout the tropics. Indeed, while there is a growing impetus to restore the islands where tropical seabirds breed [83], conservation efforts should also include their marine habitats [84] where wind plays a critical role in shaping both current and future foraging opportunities and distribution patterns. As wind patterns shift with climate change, understanding and incorporating these dynamics into marine conservation planning will be essential in ensuring effective protection.

## Data Availability

GPS tracking data available via the Seabird Tracking Database (2019 and 2022 data = datasets no. 2099 and no. 2100, respectively) [85]. Tri-axial acceleration data are at Harvard Dataverse [86]. Video data are at Harvard Dataverse [87]. Code is at Zenodo [88]. Supplementary material is available online [89].
